# The food patterns of a multicenter cohort of Brazilian nulliparous pregnant women

**DOI:** 10.1038/s41598-021-95185-2

**Published:** 2021-07-30

**Authors:** Maria J. Miele, Renato T. Souza, Iracema M. Calderon, Francisco E. Feitosa, Débora F. Leite, Edilberto A. Rocha Filho, Janete Vettorazzi, Jussara Mayrink, Karayna G. Fernandes, Matias C. Vieira, Rodolfo C. Pacagnella, José G. Cecatti

**Affiliations:** 1 Department of Obstetrics and Gynaecology, University of Campinas (UNICAMP), School of Medicine, Campinas, SP Brazil; 2 Department of Gynaecology and Obstetrics, Botucatu Medical School, Sao Paulo State University (Unesp), Botucatu, SP Brazil; 3 MEAC-Maternity School of the Federal University of Ceará, Fortaleza, CE Brazil; 4 Department of Gynaecology and Obstetrics, Federal University of Pernambuco, Recife, PE Brazil; 5 Department of Obstetrics and Gynaecology, Maternity Hospital, Federal University of Rio Grande do Sul, Porto Alegre, RS Brazil; 6 Jundiaí School of Medicine, Jundiaí, São Paulo Brazil; 7 Division of Women and Children’s Health, School of Life Course Sciences, Faculty of Life Sciences and Medicine, Kings College London, London, UK

**Keywords:** Health care, Medical research

## Abstract

Assessment of human nutrition is a complex process, in pregnant women identify dietary patterns through mean nutrient consumption can be an opportunity to better educate women on how to improve their overall health through better eating. This exploratory study aimed to identify a posteriori dietary patterns in a cohort of nulliparous pregnant women. The principal component analysis (PCA) technique was performed, with Varimax orthogonal rotation of data extracted from the 24-h dietary recall, applied at 20 weeks of gestation. We analysed 1.145 dietary recalls, identifying five main components that explained 81% of the dietary pattern of the sample. Dietary patterns found were: Obesogenic, represented by ultra-processed foods, processed foods, and food groups rich in carbohydrates, fats and sugars; Traditional, most influenced by natural, minimally processed foods, groups of animal proteins and beans; Intermediate was similar to the obesogenic, although there were lower loads; Vegetarian, which was the only good representation of fruits, vegetables and dairy products; and Protein, which best represented the groups of proteins (animal and vegetable). The obesogenic and intermediate patterns represented over 37% of the variation in food consumption highlighting the opportunity to improve maternal health especially for women at first mothering.

## Introduction

The gestational process involves a diversity of adaptations to enable mother and child to complete the entire development cycle until birth. Maternal nutrition plays a determining role in this process. Despite its complexity, maternal nutrition is the factor that can be modified^1^.

The science of nutrition has attributed the health-food relationship to an isolated nutrient, failing to consider that this reductionist approach is often unable to observe that the synergistic strength between food and nutrients has a greater influence on human health^2^. The discovery that food combination is better for health outcomes than any isolated nutrient, caused a reflection on the synergistic power between food compositions of the diet in this process. For this investigation, it is necessary to consider a hierarchical structure in nutritional analysis, initiating the exploration from the largest components (diets) to the smallest (nutrient) component. This method constitutes the “top-down” approach, which is capable of providing initial clues to further investigate nutrition^3^.

Diet is determined by a food group that is predominant in daily consumption and this food group forms the dietary patterns. Patterns may be guided by a method of hypothesis, named “a priori”, or by exploratory statistical methods using machine learning, named “a posteriori”. Dietary analyses based on the use of predefined models (a priori) are not able to assess the interrelationships between food, reducing the original information into a smaller set of food groups, with a minimal loss of information. As a result, adequacies to patterns considered “healthy or unhealthy” are measured, which do not reflect diet diversity in general which include multiple combinations of food intake^4^. Thus, when a single nutritional component is unable to reflect the health of a study group, the application of dietary patterns may provide us with relevant information about the diet, since it takes into account the high level of underlying inter-correlations^5^. From this perspective, with machine learning, it is possible to explore large volumes of data and identify profiles in the lack of a prelabelled response^6^. This study aimed to identify dietary patterns of Brazilian nulliparous pregnant women at mid-gestation, addressing the original food groups, and identify which food combinations best represents the characteristic of the choices for food consumption in this population.

## Results

**Figure 1:**
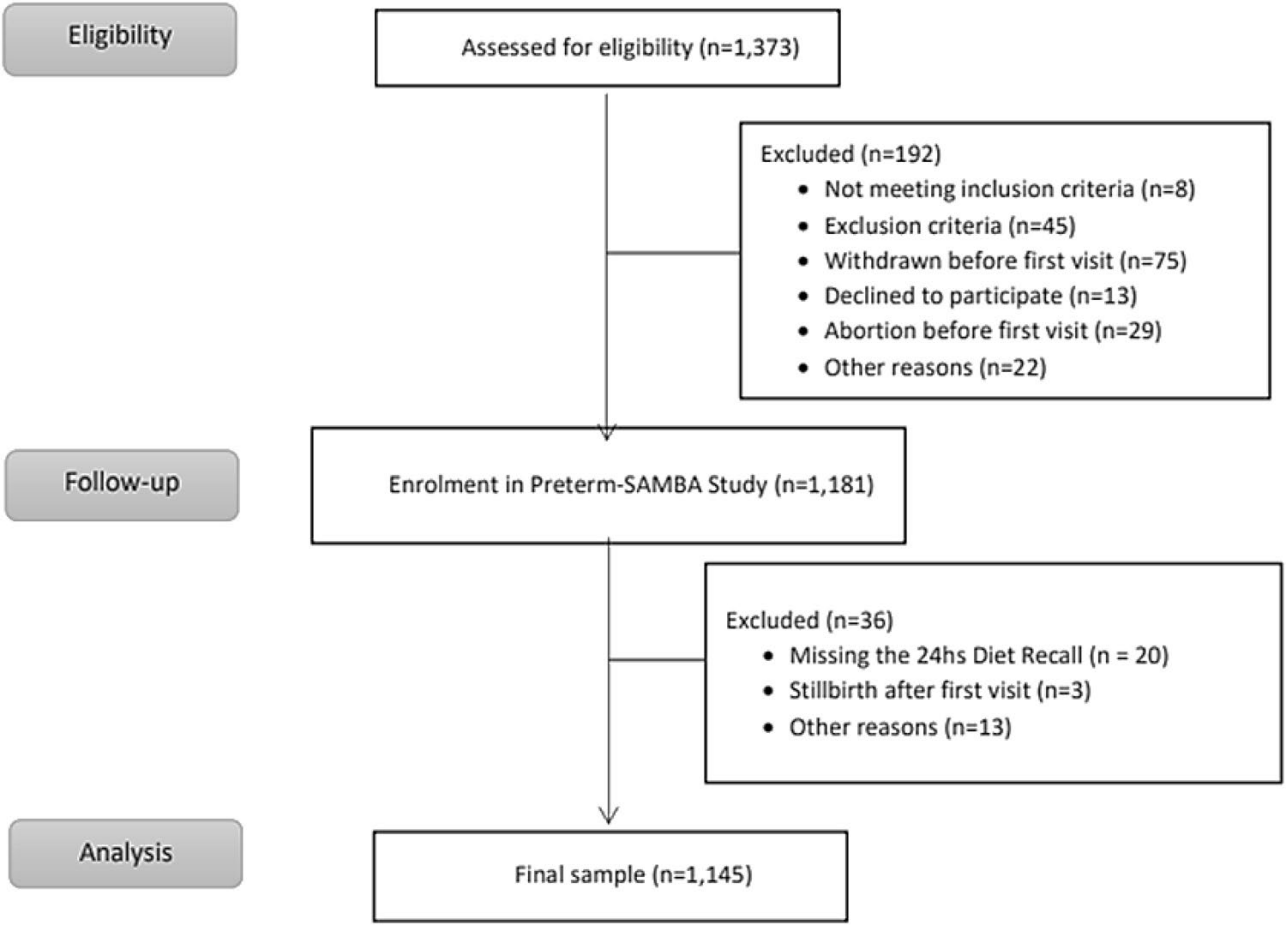
Flowchart of the sample (produced by MJ Miele).

In the 1.145 women that responded to the food recall (Fig. 1), the highest proportion of adequate BMI category was found in 40% (mean of 23.9 kg/m^2^), followed by overweight in 26% (28.3 kg/m^2^) and equal proportions of obese (35.4 kg/m^2^) and low weight (20.0 kg/m^2^) in 17% of the sample (Table 1).

**Table 1:** Sample distribution according to sociodemographic and anthropometric characteristics. *BMI* Body Mass Index measured at study entry and last visit before childbirth (Atalah, 1997).

Maternal features	N	%
**Country region**		
Northeast	556	49
South and Southeast	589	51
**Income (per year)**		
< 6,000 (U$)	47	4.1
6,000–12,000 (U$)	248	21.6
> 12,000–24,000 (U$)	375	32.7
> 24,000 (U$)	475	41.4
**Occupation**		
Paid work	579	50.5
Housewife	208	18.1
Not working*	358	31.2
**Marital Status**		
With partner	828	72.3
Without partner	317	27.6
**Maternal Color/ethnic**^**a**^		
White	442	39.0
Black	114	10.1
Brown	569	50.2
Other	8	0.7
**Maternal age (year)**		
< 20	284	24.8
20–34	779	68.0
> 34	82	7.1
**Education (year)**		
< 9	169	14.7
9–12	641	55.9
> 12	335	29.2
**BMI (study entry)**		
Low weight	196	17
Adequate	449	40
Overweight	294	26
Obese	193	17
**BMI (before childbirth)**		
Low weight	94	13
Adequate	268	36
Overweight	213	28
Obese	196	26

Table 2 shows the descriptive analysis of food groups selected according to the degree of food industrialization and calorie percentage, food groups with similar nutritional characteristics and the approximate percentage according to the mean value considered adequate for consumption.

**Table 2:** Profile distribution of food group consumption Interquartile range (IQR).

Calories (kcal)	Median (IIQ)	Total (%)
UMPF	1112.8 (748.2)	51.93
PF	590.1 (646.9)	27.54
UPF	439.9 (639.3)	20.53
**Serving (unit)**		**Adequacy (%)**
Grains	5.6 (4.3)	93.6
Fruit/vegetables	5.3 (6.6)	106.4
Dairy	1.5 (1.6)	37.5
Meat/eggs	2.7 (2.4)	133.0
Beans	3.0 (3.4)	302.0
Fat/sweets	4.5 (3.3)	226.5

*UMPF* Unprocessed and minimally processed food, *PF* processed food, *UPF* ultra-processed food, *Grains* foods rich in carbohydrates such as rice, pasta, bread, crackers, corn, potato and other tubers, *Fruits/vegetables* fruits, leaves and vegetables, *Dairy* cheese, milk and yoghurts, *Meat/eggs* animal-based protein including eggs and chicken, cow and pork meat, *Beans* foods rich in vegetable protein such as beans, soy and peas, *Fats/sweet* sugars, sweets, desserts and candies, oils, pork lard, butter and margarine. Servings considered adequate for each food group were: grains = 6, beans = 1, fruits and vegetables = 5, Meat/eggs = 2, Dairy = 4, Fat and Sweets (FS) = 2.

After dimensioning the original variables using the Principal Component Analysis (PCA) technique, five principal components were extracted (eigenvalue =  ≥ 1.03) using the Kaiser rule, which indicates that an eigenvalue of 1.0 or greater can measure the variance in all variables. Together, these five components were able to explain 81% of the variation in food consumption.

**Figure 2:**
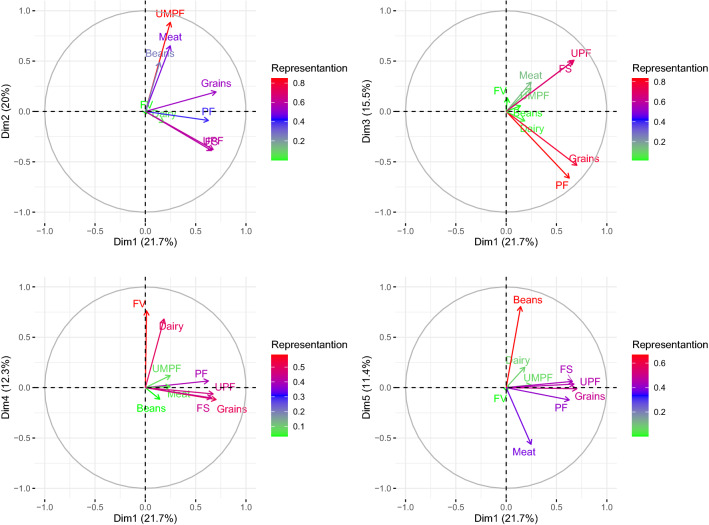
Perceptual map of interrelationships and correlations between dimensions of dietary patterns. Image obtained from the R software. *UMPF* Unprocessed and minimally processed food, *PF* processed food, *UPF* ultra-processed food, *FV* fruits and vegetables, *Meat* meat and eggs, *FS* fats and sweet.

The PCA result maximized the higher loads of variables that had the most significant influence on food consumption and decreased variables that had less influence on consumption. To assess interrelationships between variables and the five dimensions selected, rotations were made on the perceptual map, using the first component (Dim 1) as the baseline responsible for the greater variation in food consumption (21.7%) (Fig. 2). The size of the variable is displayed on a colour scale, with the higher loads represented by red and purple, and lower loads represented by blue and green vectors. The direction of the variable vectors indicates correlations between vectors and how diets are dimensioned. The angles between the vectors indicate inter-correlations between variables. The proximity between the two vectors may suggest that foods are consumed together. Factor loadings close to − 1 or 1 indicate that the variable has a strong influence on the component. Factor loadings close to 0 indicates that the variable has a weak influence on the component. The smaller angles show a dependence on consumption. In contrast, the distance observed in the map suggests greater independence, therefore we can infer that the proximity of UMPF, Beans, Meat vectors suggest a greater dependence between them, suggesting that they are consumed together (Dim2). Similarly, Dim1, UPF and FS groups and Grains and PF suggest a dependence on consumption (Fig. 2a). Concerning vector size represented by colours, the three principal dimensions (explaining 57.2% of consumption) showed the groups of Fruit/Vegetables (FV) and dairy products. It also showed that the FV group had a low correlation and size.

Figure 2b shows the representation of dispersion between the first and third dimensions, with a similar size for vectors PF, Grains and UPF, where both groups suggest a dependent consumption in both dimensions that total 37.2% of consumption. Figure 2c (Dim 1 and 4) explains 34% of variations and is the only configuration in the map where vector FV has greater participation in the diet and also indicates a shorter distance from the Dairy group, indicating dependence on consumption. Finally, in Fig. 2d with 33% of the variance, Beans are highlighted among the remaining groups followed by Grains.

**Figure 3:**
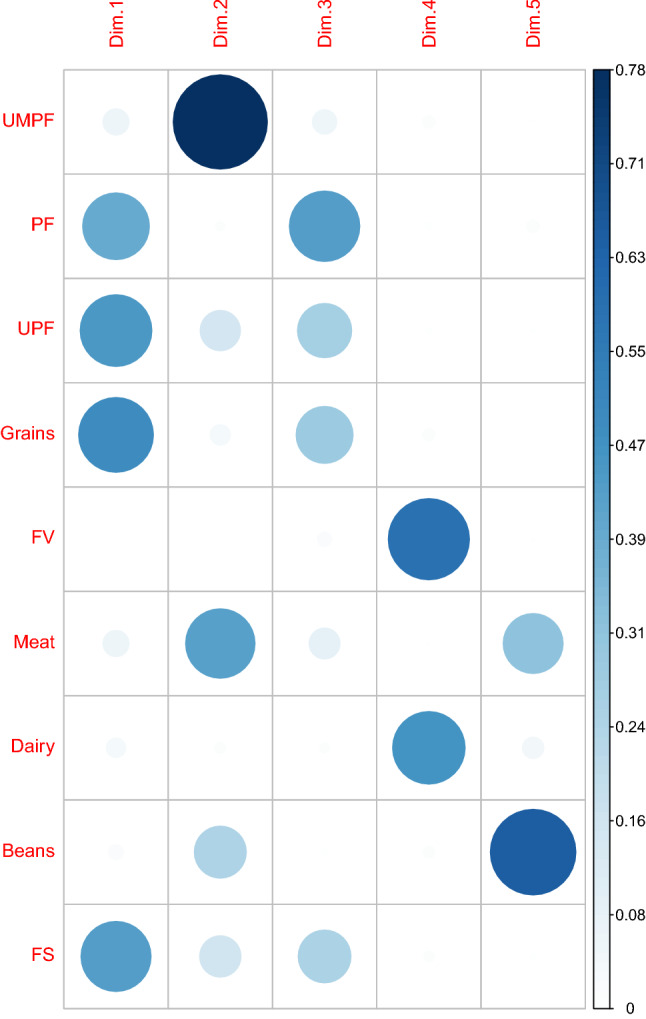
Food groups best represented in correlations of each of the five dimensions of dietary patterns. The image shows the representation of the variables loads for each component on gradual colours white to dark blue (between 0 and 0.78% on the scale). Image obtained from the R software. *UMPF* Unprocessed and minimally processed food, *PF* processed food, *UPF* ultra-processed food, *FV* fruits and vegetables, *Meat* meat and eggs, *FS* fats and sweet.

Figure 3 shows the proportions and interrelationships between each dimensional loading. Variations caused by dimensioning define the principal component combinations of the maternal diet. Considering the 5 dietary patterns (Kaiser criteria) that best represented sample variability, the diets extracted were identified according to loading characteristics and relabeled according to food group combinations: “Obesogenic”, “Traditional”, “Intermediate”, “Vegetarian” and “Protein” (Table 3).

**Table 3:** Factor loadings of food groups in the principal dietary components obtained using PCA. *PCA* principal component analysis. Result of the extraction of principal components, relabeled according to the characteristics of dietary patterns and scores.

Groups	Principal component loading				
	Obesogenic	Traditional	Intermediate	Vegetarian	Protein
UMPF	0.247	**0.886**	0.232	0.120	0.027
PF	**0.627**	− 0.089	− **0.662**	0.066	− 0.123
UPF	**0.674**	− 0.378	**0.509**	− 0.062	0.035
Grains	**0.703**	0.194	− **0.535**	− 0.118	− 0.011
Fruits/vegetables	0.012	− 0.023	0.135	**0.763**	− 0.036
Meat/eggs	0.247	**0.652**	0.291	0.011	− **0.563**
Dairy	0.186	− 0.108	− 0.095	**0.681**	0.203
Beans	0.143	**0.488**	0.057	− 0.115	**0.805**
Fats/sweets	**0.658**	− 0.388	**0.496**	− 0.102	0.062
Eigenvalue variance	**21.7**	**20.0**	**15.5**	**12.3**	**11.4**
Cumulative eigenvalue	**21.7**	**41.7**	**57.2**	**69.5**	**80.9**

In boldface, loads ≥ 0.40 (negative and positive), contribute to the creation of each component.

*UMPF* Unprocessed and minimally processed food, *PF* processed food, *UPF* ultra-processed food, *Grains* foods rich in carbohydrates. *Fruit/Vegetables* fruits, leaves and vegetables, *Dairy* cheese, milk and yoghurt, *Meat/eggs* eggs and animal-derived meat, *Beans* beans, soy and peas, *Fats/Sweets* fats and sugar.

Eigenvalue variance represented the percentage of each diet pattern and cumulative represents the sum of eigenvalues of dietary explanation.

## Discussion

In our study, it was observed a low frequency in the consumption of dairy and high consumption of UPF among Brazilian nulliparous women at mid-pregnancy. Results comparing data of the Brazilian National Survey of 2002/2003 to 2018/2019, revealed a decrease of dairy intake of up to 42% and an increase of ultra-processed food, representing 18.4% consumed at home eating^7^. Also, consumption of FS and Beans was higher than recommended adequate levels. The use of PCA uncovered 5 dietary patterns that explained 81% of food consumption variation amongst nulliparous women in Brazil. There are two components thought to have a bad influence on health (obesogenic and intermediate diet) which represented 37.2% of the variation, suggesting there is potential to improve women’s food choices.

Our results were not different than those from other studies about the UPF levels intake in Brazil, but considerably lower than in the USA. In a study of an American adult population, the high consumption of ultra-processed foods accounted for 58% of calories ingested. Of these, 89% were composed of sugar and candies, which lead to weight gain, especially in women^8^. Whereas, in a sample of 8977 Brazilian adults, the consumption of UPF has a positive association with the increases in BMI. The mean observed was 22.7% of the total energy of UPF^9^. The data from high and middle-income countries, revelated UPF products leads the food system from retailing to food services. In Brazil, the rise of UPF consumption had increased sharply, jumped from 18.7% in 1987 to 26.1% in 2003^10^. A study with Brazilian pregnant women revealed a quarter of the total energy intake was provided from UPF, corresponding to 19.3% of total energy intake between the first and second trimester^11^. Although there is no determination about the adequate amount of UPF consumption, there is a consensus about as lower as possible to prevent unhealthy consequences^12,13^.

Nevertheless, changes in dietary patterns have revealed the consumption of unhealthy items throughout the world, indicating a generally worse quality of the human diet^14^. A study evaluating a diet “a posteriori” in a group of 63,808 Norwegian pregnant women obtained as the result of the principal component a diet rich in vegetables, fruits and whole cereals and low consumption of processed meat and sugar^15^. The use of specific exploratory techniques to identify dietary patterns a posteriori in pregnant women is still rare.

In our study, we observed a substantial increase in BMI for the groups of women classified as obese and overweight. In addition, we highlight the importance and originality of our study regarding the gestation period evaluated and the characteristics of the sample formed by nulliparous pregnant women.

This is a daunting prospect since this is a particular group of nulliparous pregnant women that may have another pregnancy. Post gestational weight retention in obese or overweight women increases the risks for diabetes during a second pregnancy, while weight loss reduces this risk^16^. On the other hand, nutritional counselling on dietary patterns can reduce excess weight during pregnancy, as well as post gestational weight retention and adverse outcomes for the mother and offspring^17^. Two other previous studies performed with the same sample of pregnant women showed a positive association between obese/overweight women and the development of pregnancy-related diseases such as gestational diabetes. Obese women who had more weight gain had a higher incidence of preterm delivery^18,19^.

On the other hand, the greater intake of UMPF, Fruit and Vegetables is to be considered. The benefit of diets rich in dairy products, fruits and vegetables resulted in greater benefits, substantially reducing pro-inflammatory biomarkers, hypertensive events, dyslipidaemias and better blood glucose control, decreasing insulin resistance, in addition to better weight control^20^. Our results have shown that patterns except for the “Vegetarian” diet explained by 12.3%, the lack of consumption of dairy foods, fruits and vegetables was frequently absent in the three pattern diets which together have explained > 57% of dietary identified. These findings also reinforce the need to observe dietary habits in pregnant women and the opportunity to improve health by promoting adequate counselling. The results showed the variability of foods that were consumed together and pointed to a trend in eating behaviour. The food group restriction as vegetables and fruits and replacement of homemade meal consumption by the incorporation of industrialized meals, with amounts of sugar and trans-fat, reinforce our hypothesis that dietary patterns should be investigated to gain knowledge and understanding of the diet^5,21^. A reflection about food synergism exposes the complexity of human eating habits. There is no such thing as a “perfect model” or a “static” diet without changes in real-life models and therefore new eating habits. A careful and constant evaluation is recommended^22^.

This study did not aim to present a definitive response to Brazilian dietary patterns during pregnancy, owing to its exploratory nature. Nevertheless, the daily eating reality of these women was outlined in the profile description found in the study. There is no perfect dietary pattern. All components identified in our analysis are needed in different quantities for a balanced diet. The binary assessment of a person overall diet as ‘healthy’ or ‘unhealthy’ does not account for complexities such as having increased consumption of both, a component that has a good influence on the health but also a component that has bad influence^23^. Furthermore, using adequacy scores for calories or nutrients is useful for evaluation, although it is important to observe numerous biochemical interactions triggered by food in the human body. A broader analysis of this topic is required^24^.

Individuals choose food, not nutrients. Food choice is based on affective memory, specific flavours of regional cuisine, emotions from different stages of human life, and the “practicality” of a ready-made meal. All these compositions suffer constant changes in diets and habits. Industrialized food has become a more frequent meal option worldwide^25^. Although we had over one thousand diet recalls, we also have a limitation of just one questionnaire per woman. However, this was an exploratory study, which found a combination of foods that can develop or aggravate pregnancy-related conditions. Moreover, these dietary patterns are from the 2nd trimester when the women no longer suffer important influences from the pregnant state as nausea events. In addition, theoretically, with this early identification of inappropriate dietary patterns in mid pregnancy among overweight/obese women, possibly interventions to modify this could have been introduced, what would make a single recall insufficient to detect such changes. This could also be considered a bias of tool assessment, however the proportion of pregnant women followed in the cohort that were considered as overweight/obese only increased along gestation, suggesting no effective interventions were in fact implemented. Concurrent, we still have half of the pregnancy ahead, one reasonable time to offer information and encourage changes in habits. The differential of this study was that it evaluated a sample with culinary diversity from different country regions, to observe and describe which compositions were formed, differentiating food characteristics and degree of industrialization.

This study confirms previous inadequacies in maternal food consumption in Brazil. In addition, it describes the variability in dietary patterns of this sample. Variation in components of eating patterns can be seen as an opportunity to influence eating behaviour and should be considered when developing eating educational programs. Lifelong physical activity and dietary education before women become pregnant could contribute to the improvement of overweight and obesity. However, if pregnancy is a short period of time, halfway through pregnancy is even shorter. Still, there is time to apply guidelines that can help, through diet, to reduce risks and prevent excessive weight gain and dietary disorders.

## Methods

### Study design and setting

This is an analysis of secondary objectives of a multicentre cohort of nulliparous pregnant women from the primary study termed “Preterm-SAMBA—Preterm Screening and Metabolomics in Brazil and Auckland”^26^. It is a cross-sectional approach nested in a cohort study. The study included women between July 2015 and July 2018 in five maternity units from three country regions (Southeast, South and Northeast) that provide care as part of the Brazilian public health system. Nulliparous women with a single fetus and gestational age between 19 and 21 weeks were invited to participate in the study. Pregnancy was confirmed and dated by an early ultrasound scan. Nulliparous women without associated comorbidities, with single gestation and gestational age between 19 and 21 weeks were invited to participate in the study with the purpose of obtaining a low-risk population of pregnant women not influenced by severe clinical conditions or high parity. Furthermore, the regular use of steroids, aspirin, heparin, calcium supplementation, fish oil, vitamin C, or vitamin E, were considered as excluded criteria for this study. Other vitamins as iron and folic acid supplements, routines in prenatal care, or polivitamins were not exclusion criteria. The dietary patterns addressing the original food groups were evaluated from the food recall questionnaire applied during the first original study visit. The flowchart of the sample is presented in Fig. 1.

### Dietary assessment

This study was approved by the Institutional Review Boards of the University of Campinas and by those of all participating centres (coordinating centre protocol 20182318.8.0000.5404), in addition to the Brazilian National Ethics Committee for Research (CONEP).

To explore the dietary profile of women, we used the concept of Willett^27^ where the 24 h-food recall was applied once during the first visit of the study, between 19 and 21 weeks of gestation, on different days of the week (including the analysis of food intake also on weekends) as being sufficient for this purpose. Dietary analysis was conducted by a specialized dietitian using the multi-passage method^28^. To improve the understanding of serving sizes and determine the amount of food consumed, an album of images containing photographic records of household measures and food sizes were used during the interviews with participating women^29^.

To standardize servings, the household measures reported were converted into grams or millilitres. The Brazilian consumption manual was the reference^30,31^. To ensure that amounts were in accordance with consumption, information on labels of industrialized foods and typical recipes of Brazilian cuisine were extracted and reproduced by national Food Tables.

The DietWin-Plus Brazilian software (version 3090, https://dietwin.zendesk.com/hc/pt-br/categories/200182303-Dietwin) was used for dietary analysis, allowing the choice to use national and international Food Tables^32–34^, in addition to the inclusion of recipes and industrialized food labels.

Using the “xlsx”, “tidyverse” and “magrittr” packages of R Software (R version 3.6.3), data of the 1.145 food recalls were treated and reorganized into a single document for analysis, resulting in a single list with all crude data of consumption in weight, volume and calories. From this list, foods were categorized into groups according to nutritional characteristics based on dietary recommendations in the Food Guide for the Brazilian population^35^, such as (1) foods rich in carbohydrates in general, including potatoes and other tubers, bread, crackers, pasta, rice, cereals, corn and cornmeal, composing the “Grain” group; (2) vegetables, leaves and fruits, were grouped forming a single group called “Vegetables and Fruits”; (3) cheese, milk and yoghurt formed the “Dairy” group; (4) animal-derived proteins such as meat (chicken, bovine and pork) and eggs formed the “Meat and Eggs” group”; (5) foods rich in vegetable protein such as beans, soya and peas formed the family of legumes labelled the “Beans” group; (6) foods such as sugars, sweets, desserts and candies, oils, butter, pork lard and margarine form the “Fat and Sugar” group. Servings were established according to the calorie content of the food group (serving/calorie), from the national food consumption tables converted into household measures^30,36^. The mean servings considered adequate for each food group was based on the Brazilian Food Pyramid Guide^37^. Finally, the listed foods were also categorized based on the degree of industrial processing, according to the NOVA Food Guide^38^. In this study, three categories were separated: (1) “unprocessed food or minimally processed food”; (2) “processed food”, (3) “ultra-processed food”. Added salt, fat, and sugar corresponding to the NOVA Guidelines were incorporated into meal preparation.

The height and weight of the pregnant woman were measured in the first prenatal visit and the Body Mass Index (BMI) was calculated, generated by software using the formula weight/height^2^ (kg/m^2^). To perform the analysis of BMI categories, the Atalah Index according to the week of gestation was used (19–21 weeks)^39^. Analysis of sociodemographic data was obtained through a self-reported questionnaire. Classifications were reported according to the responses obtained for annual family income converted into categories corresponding to American dollars: (1) up to 6 thousand; (2) between 6 and 12 thousand; (3) between 12 and 24 thousand; (4) Above 24 thousand. Women responded whether they had a paid job outside the house or worked at home doing domestic work without remuneration, and how long they attended school: less than 12 years or ≥ 12 years of study. Skin colour was categorized as black or other. All evaluations were made by previously trained healthcare professionals and data was inserted in real-time into the electronic platform of the study (MedSciNet AB, Sweden, https://medscinet.com/).

### Statistical analysis

Analysis of sociodemographic data was conducted according to records and the percentage representation in whole data (n%). Dietary assessment was made by using the original data, initially to check data normality, creating a histogram followed by a normality test (Shapiro–Wilk) to evaluate data distribution in food groups. A descriptive analysis using the median and interquartile range was conducted for the initial exploration of food consumption. Then a comparison was made according to the degree of food industrialization and total calories and approximation of the number of servings per food group with the mean serving recommended (%).

Afterwards, multivariate data analysis was conducted by the Principal Component Analysis (PCA) technique, with Varimax orthogonal rotation^40^. It is an exploratory analysis technique that identifies new dietary patterns using original data. These patterns are named principal components. The original data was used to construct an axial system, where the linear relations of covariance and correlation in different dimensions were measured. The technique reduces the number of original variables and evaluates food groups that are consumed together. Furthermore, the result can explain the variability in food consumption that best reflected the dietary pattern of the sample. For this analysis, the original data was initially standardized by transforming calorie measurements and food servings in a homogenous scale for analysis through the z-score. To determine the adequate number of the principal components, the Kaiser rule was used with a cut-off on the eigenvalues of ≥ 1.0, along with the analysis of the proportion of variance explained by the Scree Plot graph (Cattell test)^41^. To determine the creation of each principal component that depicts the dietary patterns found, a cut-off value of ≥ 0.40 (positive or negative) was adopted for loading scores, as well as to explain the distribution of variables. From an exploratory perspective, purely technical criteria may help in making this decision. The result depicted the characteristics of principal components that best represented the combined consumption of food groups. These food groups were relabelled according to the characteristics of dietary combinations. For data exploration and statistical analysis, the ‘‘FactoMineR”, “factoextra”, “cluster” and “corrplot” packages of software R (version 3.6.3)^42^ were used.

### Ethics declarations

All women signed an individual two-way informed consent form before study admission. The Preterm-SAMBA study was conducted, in compliance with the Declaration of Helsinki (2013), following national and international regulations according to the Brazilian Resolution CNS 466/12. It was approved by the Institutional Review Boards of all participating centres (coordinating centre protocol 20182318.8.0000.5404), in addition to the National Ethics Committee for Research (CONEP). All women included in this study signed an individual informed consent term, before admission. This manuscript follows the guidelines of the Strengthening the Reporting of Observational Studies in Epidemiology (STROBE)^43^.

## Data Availability

Data are available upon reasonable request.
